# The Common Task

**Published:** 1907-11-02

**Authors:** 


### THE COMMON TASK.

**Correspondence and Queries** for this section should be sent to the Editor of The Hospital, 28 Southampton Street, Strand, London, and marked "Nursing Administration."

### The Out-patient Staff.

One of the difficulties confronting the matron of small hospitals is that of isolating her out-patient staff altogether from the ward work. In clinical hospitals generally, and in all institutions with large out-patient departments, there is as a matter of course a special sister attached to this branch of work, with one or more probationers under her, and the work is of so absorbing a character that their whole time is engaged. But in small hospitals the out-patient work is not always sufficient to absorb the whole time of even one nurse. Looking down the list of hospitals scheduled in Burdett's "Annual" under the head of the Nursing Department, instances may be observed in which no nurses are exclusively engaged in the out-patients' and receiving room. This means that the nurses who perform the out-patients' work are taken from the wards, and fit in their duties accordingly. A careful matron would not, of course, admit any interchange of this kind between the surgical or gynæcological wards. But when the nurse returns even to the medical wards after a morning spent in receiving an indiscriminate throng of persons suffering from untabulated diseases, she exposes the patients to very grave risk. Almost every form of infectious disease finds its entrance in the early stages into the out-patients' department, and many complaints, such, for instance, as diphtheria, may be at the outset indistinguishable and yet highly contagious. The risk would be minimised if the out-patient nurse were employed in the office and home departments, and this would seem to be the best solution of the difficulty.

### Jealousy in Midwives.

The curious attitude of suspicion which characterises many midwives in their dealings with their inspectors, and with other servants of the public, such as the medical officer of health and the health visitors, is a survival of former days, when the midwife, instead of being a qualified person with official sanction to practise her calling, was merely in the position of a charlatan, and hence was prone to regard herself as one whose hand was against every man. As time goes on the certified midwife will certainly become accustomed to her dignity as a member of a valuable and ancient order, and will welcome rather than distrust the visits of those who are appointed to keep up a high standard of efficiency in the calling. We have not heard of any instance in which the inspector has aimed at confounding those in her jurisdiction by petty complaints. These officials are generally overwhelmed with work, and are commonly but grudgingly received by those who should reckon them their best friends. We know of one instance in which the midwife refused to reply to any of the ordinary questions addressed to her, remaining disconcertingly mute, while the gentle lady charged with the duty of superintending her work got through such inspection as was possible. In general it may be safely concluded that such suspicious distrust springs from some conscious breach of regulations, and that this is not invariably the case, but is affected even by conscientious women, arises from the wrong-headed attitude into which so many midwives have allowed themselves to be drawn.

### Old Style and New Style.

It is seldom that the old and the new can be studied side by side to such advantage as at the present moment in the Royal Portsmouth Hospital. While the principal wards here are of modern construction, having been rebuilt within the last few years, the children's wards are as they were in the infancy of the institution, and the contrast is truly deplorable. In place of hard floors, lofty ceilings, and generous windows, the two children's wards at the top of the building present a dingy flooring of old boards, and can boast of but four small windows apiece, set too high for light and too low for ventilation. We understand that only lack of funds stands in the way of the entire remodelling of this ancient part of the hospital, as the architect has left matters so arranged that a new wing could be commenced to-morrow. This is the only children's hospital available for the large towns of Portsmouth and Southsea, and we think if people with money in their pockets could be induced to visit the wards the crying need would soon be met. Brightness, sunshine and air, so important in the treatment of all patients, assume in the case of children paramount importance. It speaks much for the determination of the nursing staff that the wards, though destitute of every advantage as regards construction, have still a well-cared-for appearance. Construction defects certainly serve to put the staff on their mettle, but all the same Portsmouth and Southsea ought to see to it that an immediate effort is made on the children's behalf.

---

THE HOSPITAL *November* 2, 1907.

